# Energy Decline May Predict Mild Parkinsonian Signs in Community-Dwelling Older Adults

**DOI:** 10.1093/geroni/igab046.701

**Published:** 2021-12-17

**Authors:** Rebecca Ehrenkranz, Qu Tian, Andrea Rosso, Nancy W Glynn, Lana Chahine, James Hengenius, Xiaonan Zhu, Caterina Rosano

**Affiliations:** 1 University of Pittsburgh Graduate School of Public Health, Pittsburgh, Pennsylvania, United States; 2 National Institute on Aging, Baltimore, Maryland, United States; 3 University of Pittsburgh, Pittsburgh, Pennsylvania, United States

## Abstract

Mild Parkinsonian Signs (MPS) are common in older adults without overt neurological disease. MPS are often progressive and predict disability and dementia, yet little is known about predictors for MPS. Low self-reported energy is associated with mobility impairment, which is a hallmark of MPS. Yet whether self-reported energy relates to MPS is unknown. We explored the association of changes in self-reported energy with MPS in 293 participants (aged 83 ± 2.8 years, 58% women, 61% White) free of dementia and Parkinson’s Disease in the Health, Aging and Body Composition Study. Self-reported energy was assessed on a 0-10 scale annually between Year 2 and Year 10 (mean follow-up: 8 years) and its slope was estimated via linear mixed effects models. MPS were evaluated at Year 10 based on the Unified Parkinson Disease Rating Scale motor component. On average, self-reported energy declined 0.06 points per year. In a linear regression model adjusted for age, fatigue, and comorbidities, those with MPS had steeper SEL decline (β [Standard Error] = -0.358 [0.119]) in the prior eight years than those without MPS. Thus, declining self-reported energy may be a risk factor for MPS. Self-reported energy is easily evaluated in routine clinic visits, and may be a modifiable risk factor that can be targeted to reduce the incidence of MPS.

